# Young Adult Physical Activity Trajectories and Midlife Nonalcoholic Fatty Liver Disease

**DOI:** 10.1001/jamanetworkopen.2023.38952

**Published:** 2023-10-20

**Authors:** Junia N. de Brito, Daniel J. McDonough, Mahesh Mathew, Lisa B. VanWagner, Pamela J. Schreiner, Kelley Pettee Gabriel, David R. Jacobs, James G. Terry, John Jeffrey Carr, Mark A. Pereira

**Affiliations:** 1 Department of Family Medicine and Community Health, University of Minnesota Medical School, Minneapolis; 2 Division of Epidemiology and Community Health, University of Minnesota School of Public Health, Minneapolis; 3 Division of Digestive and Liver Diseases, University of Texas Southwestern Medical Center, Dallas; 4 Department of Epidemiology, University of Alabama at Birmingham School of Public Health, Birmingham; 5 Department of Radiology, Vanderbilt University Medical Center, Nashville, Tennessee

## Abstract

**Importance:**

Physical activity (PA) is recommended for preventing and treating nonalcoholic fatty liver disease (NAFLD). Yet, how long-term patterns of intensity-based physical activity, including moderate-intensity PA (MPA) and vigorous-intensity PA (VPA), might affect the prevalence of NAFLD in middle age remains unclear.

**Objective:**

To identify distinct intensity-based PA trajectories from young to middle adulthood and examine the associations between PA trajectories and NAFLD prevalence in midlife.

**Design, Setting, and Participants:**

This population-based cohort of 2833 participants used the Coronary Artery Risk Development in Young Adults study data. The setting included field clinics in Birmingham, Alabama; Chicago, Illinois; Minneapolis, Minnesota; and Oakland, California. Data analysis was completed in March 2023.

**Exposures:**

PA was self-reported at 8 examinations over 25 years (1985-1986 to 2010-2011) and separately scored for MPA and VPA.

**Main Outcomes and Measures:**

NAFLD was defined as liver attenuation values less than 51 Hounsfield units after exclusion of other causes of liver fat, measured using computed tomography in year 25 (2010-2011).

**Results:**

Among a total of 2833 participants included in the sample, 1379 (48.7%) self-identified as Black, 1454 (51.3%) as White, 1206 (42.6%) as male, and 1627 (57.4%) as female from baseline (1985-1986) (mean [SD] age, 25.0 [3.6] years) to year 25 (2010-2011) (mean [SD] age, 50.1 [3.6] years). Three MPA trajectories were identified: very low stable (1514 participants [53.4%]), low increasing (1096 [38.7%]), and moderate increasing (223 [7.9%]); and 3 VPA trajectories: low stable (1649 [58.2%]), moderate decreasing (1015 [35.8%]), and high decreasing (169 [6.0%]). After adjustment for covariates (sex, age, race, study center, education, smoking status, and alcohol consumption), participants in the moderate decreasing (risk ratio [RR], 0.74; 95% CI, 0.54-0.85) and the high decreasing (RR, 0.59; 95% CI, 0.44-0.80) VPA trajectories had a lower risk of NAFLD in middle age, relative to participants in the low stable VPA trajectory. Adjustments for baseline body mass index and waist circumference attenuated these estimates, but the results remained statistically significant. The adjusted RRs across the MPA trajectories were close to null and not statistically significant.

**Conclusions and Relevance:**

This cohort study of Black and White participants found a reduced risk of NAFLD in middle age for individuals with higher levels of VPA throughout young to middle adulthood compared with those with lower VPA levels. These results suggest the need for promoting sustainable and equitable prevention programs focused on VPA over the life course to aid in lowering NAFLD risk.

## Key Points

**Question:**

Are 25-year intensity-based physical activity trajectories associated with nonalcoholic fatty liver disease (NAFLD) in midlife?

**Findings:**

In 2833 Black and White participants in the Coronary Artery Risk Development in Young Adults cohort study, those in a high decreasing vigorous-intensity physical activity (VPA) trajectory had a 41% lower risk of NAFLD than those following a low stable VPA over 25 years. No associations were found between moderate-intensity physical activity trajectories and NAFLD.

**Meaning:**

These findings suggest the importance of sustained VPA from early adulthood to midlife for lower NAFLD risk, and the importance of equitable prevention programs that promote lifelong participation in vigorous-intensity activities.

## Teaser

This cohort study investigates intensity-based physical activity (PA) trajectories from young to middle adulthood and examines the associations between PA trajectories and nonalcoholic fatty liver disease prevalence in midlife.

## Introduction

Nonalcoholic fatty liver disease (NAFLD) is the most common cause of chronic liver disease worldwide. In the United States, the prevalence of NAFLD among adults is approximately 31%,^1^ with important racial and ethnic disparities in NAFLD prevalence.^2^ Individuals living with obesity, hyperlipidemia, metabolic syndrome, type 2 diabetes, and hypertension are more likely to develop NAFLD,^3^ which are all known risk factors for cardiovascular diseases.^4^ Changes in weight and lifestyle modifications, including dietary intake and physical activity (PA), are currently the primary therapeutic recommendations for managing NAFLD.^5^ Thus, it is important to improve our understanding of how to prevent NAFLD through modifying lifestyle risk factors.

Systematic reviews of randomized and nonrandomized clinical trials investigating the effects of PA on NAFLD indicated that PA resulted in significant therapeutic benefits on NAFLD, including a reduction in intrahepatic lipids independently of weight loss.^6,7,8^ Moreover, different forms of PA (eg, aerobic, resistance) seem to have a beneficial effect on NAFLD.^8^ However, little is known about how long-term patterns of different forms of PA (eg, moderate-intensity and vigorous-intensity) might affect NAFLD prevalence in middle age. By identifying patterns of PA over time, the delivery of programs aimed at modifying lifestyle risk factors can be tailored to patients at greater risk for NAFLD.

In this study, we aimed to (1) identify distinct PA trajectories from young to middle adulthood over 25 years and (2) examine the associations between intensity-based PA trajectories and NAFLD prevalence in middle age (ages 43-55 years) in a racially diverse population of adults. We hypothesized that multiple PA trajectory patterns exist within the Coronary Artery Risk Development in Young Adults (CARDIA) population and that, when considering a specific PA intensity (eg, VPA), participants in a trajectory of higher levels of PA over time would be associated with a lower likelihood of NAFLD in middle age compared with those in a trajectory of lower levels of PA.

## Methods

### Study Design and Sample

The CARDIA study is an ongoing prospective observational cohort designed to investigate risk factors for cardiovascular disease. The CARDIA study design, sampling strategy, and methods have been described elsewhere.^9^ Briefly, in 1985 to 1986, 5115 self-identified Black and White men and women aged 18 to 30 years (year 0: Y0) were recruited from 4 US urban sites: Birmingham, Alabama; Chicago, Illinois; Minneapolis, Minnesota; and Oakland, California. Participants have been followed up for more than 35 years with in-person examinations and detailed collection of sociodemographic and clinical data at Y0, Y2 (1987-1988), Y5 (1990-1991), Y7 (1992-1993), Y10 (1995-1996), Y15 (2000-2001), Y20 (2005-2006), Y25 (2010-2011), Y30 (2015-2016), and Y35 (2020-22). Retention rates have been high among survivors throughout the 30 years of completed follow-up.^10^ All study protocols were approved by the institutional review boards at all study sites, and all participants provided informed consent at each examination. This study followed the Strengthening the Reporting of Observational Studies in Epidemiology (STROBE) reporting guideline for cohort studies.

A total of 3181 participants had liver attenuation measured at Y25. Of those with liver attenuation data, we excluded a total of 331 participants as follows: those with self-reported cirrhosis or viral hepatitis (n = 53); risk factors for chronic liver disease (eg, intravenous drug use [n = 51]) or causes of secondary hepatic steatosis (ie, alcohol consumption of at least 14 drinks per week in women and at least 21 drinks per week in men [n = 152]), HIV (n = 27); and medications known to cause hepatic steatosis (eg, valproic acid, methotrexate, tamoxifen, steroids, amiodarone) (n = 48). After these exclusions, the resulting sample with liver attenuation data was 2850. An additional 17 exclusions were made for participants who did not have at least 3 PA measures during follow-up. The final analytic sample size was 2833 participants.

### Measures

#### PA Assessment

Participants reported PA at each examination using the interviewer-administered CARDIA PA questionnaire.^11^ Participants were asked for their frequency of habitual activity during the past 12 months in 13 categories (8 vigorous intensity and 5 moderate intensity) of recreational sports, leisure, and occupational activities. Using a computer-based algorithm, total PA scores were calculated based on frequency (number of months), intensity score of the activity (3-8 metabolic equivalents [METs]), and a weighting factor to correspond to the duration of each activity (2-5 hours per week).^11^ PA scores were expressed in exercise units (EU), with separate scores for moderate-intensity PA (MPA) and vigorous-intensity PA (VPA). Total PA scores were the sum of scores for all activities. A total of 300 EU is approximately equivalent to 150 minutes of MPA or 75 minutes of VPA per week, as recommended by the 2018 Physical Activity Guidelines for Americans.^12,13^ The CARDIA PA questionnaire has validity and reliability comparable with other PA questionnaires.^11,14^ We included PA data collected at 8 examinations (Y0, Y2, Y5, Y7, Y10, Y15, Y20, and Y25) over 25 years.

#### Hepatic Steatosis Assessment

Noncontrast computed tomography (CT) measured liver fat at Y25. Scans of the abdomen were performed using multidetector CT scanners as previously described.^15^ Quality control and imaging analyses were performed at a core reading facility (Wake Forest University Health Sciences, Winston-Salem, North Carolina). Liver attenuation (LA) measured in Hounsfield units (HU) was used as the CT diagnostic criteria for hepatic steatosis. In this study, any NAFLD was defined as LA of less than 51 HU (primary outcome) and moderate-to-severe NAFLD (>30% steatosis) as LA less than or equal to 40 HU (exploratory outcome) after exclusion of other causes of liver fat.^16^ An LA cutoff of less than 51 HU approximates a liver-to-spleen ratio less than 1.0 and indicates at least mild steatosis is present. CT slices of the upper abdomen were performed to measure LA. The mean of 9 measurements in the right lobe of the liver on 3 distinct CT slices was used to determine mean LA values. In this cohort, the quantitative measurement of LA relied on a workflow within the National Institutes of Health’s Center for Information Technology Medical Image Processing, Analysis, and Visualization application.^15^ Trained readers avoided measuring regions, including large hepatic vessels and focal hepatic lesions.^17,18^ On a randomly selected sample of 156 participants, the interclass correlation coefficient between different readers was high for LA (0.975).^15^

### Statistical Analysis

Group-based trajectory modeling (GBTM)^19^ with a censored normal distribution was used to identify PA trajectory groups from early to middle adulthood (n = 2833). GBTM is a specialized application of finite mixture modeling. It provides a flexible approach for identifying distinctive clusters of individuals following similar developmental trajectories within a given population.^19^ To identify the best-fitting trajectories, we used the bayesian information criterion (best models have smaller bayesian information criterion), proportion of participants in each trajectory (≥5% of total sample), mean posterior probability of group membership (near 1.0) (eTable 1 in Supplement 1), and the shape of the observed trajectories.^19^ The trajectory groups were named based on the qualitative examination of the visual pattern of change in PA during the 25 years of follow-up. We labeled trajectories based on the 300 EU threshold: below as low or very low, approximately 300 EU as moderate, and above as high.

Descriptive statistics summarized Y0 and Y25 sample’s characteristics by PA trajectory groups. Categorical variables were presented as frequency (percentage), whereas continuous variables were presented as mean (SD). The associations between the PA trajectory groups and NAFLD were examined using modified Poisson regression with robust SEs.^20,21^ Models estimated risk ratios (RR) and 95% CI.^21^ This approach was chosen over conventional methods for binary outcomes (logistic regressions) because the prevalence of NAFLD exceeded 10%, thus estimates were reported using RR instead of odds ratios.^21,22^ Clinically important covariates were chosen a priori for inclusion in multivariable models given their potential confounding roles. Models were sequentially adjusted for baseline sex assigned at birth (male or female), self-reported race (Black or White according to CARDIA’s study design), study center, and Y25 age, years of education, and lifestyle risk factors (smoking status [yes or no], diet quality score^23,24^ measured at Y20, and alcohol consumption). Body mass index (BMI; calculated as weight in kilograms divided by height in meters squared) and waist circumference measured at baseline were separately added to the fully adjusted model (ie, after adjusting for all other potential confounders). Because we hypothesized that BMI and waist circumference measured at Y25 were on the causal pathway between PA trajectories and NAFLD, these variables were not included as covariates. We also explored whether race and sex were effect modifiers. All covariates had less than 1% missing data, except for diet quality score (21% missing). We verified linearity assumptions for continuous covariates through visual inspection, quadratic terms, and quartile categorization. Two-sided *P* < .05 was considered statistically significant. All analyses were performed in Stata 17.0 MP (StataCorp) from January to March 2023.

## Results

### Participant Characteristics

Among a total of 2833 participants included in the sample, 1379 (48.7%) self-identified as Black, 1454 (51.3%) as White, 1206 (42.6%) as male, and 1627 (57.4%) as female from baseline (1985-1986) (mean [SD] age, 25.0 [3.6] years) to Y25 (2010-2011) (mean [SD] age, 50.1 [3.6] years). Table 1 presents participants’ selected baseline and Y25 characteristics by MPA and VPA trajectories. At baseline, participants in the very low stable MPA trajectory had a greater proportion self-identifying as Black and female and engaging in lower VPA levels than participants in the low stable and moderate increasing PA trajectory groups. Participants in the low stable VPA trajectory group predominantly self-identified as Black, female, currently smoking, and engaging in lower MPA levels than participants in the high decreasing trajectory group. There were 681 participants (24.0%) who met the criteria for NAFLD at LA less than 51 for MPA trajectories, and there were 681 (24.0%) for VPA trajectories.

**Table 1.** Selected Baseline (Year 0) and Year 25 Participant Characteristics by MPA and VPA Trajectory Groups (N = 2833)

Characteristics^a^	Mean (SD)					
	MPA trajectories			VPA trajectories		
	Very low stable (n = 1514)	Low increasing (n = 1096)	Moderate increasing (n = 223)	Low stable (n = 1649)	Moderate decreasing (n = 1015)	High decreasing (n = 169)
**Baseline (Year 0)**						
Age, y	24.8 (3.7)	25.1 (3.5)	25.1 (3.5)	25.0 (3.7)	25.0 (3.5)	24.9 (3.2)
Race, No. (%)						
Black	896 (59.2)	418 (38.1)	65 (29.1)	890 (54)	413 (40.7)	76 (45)
White	618 (40.8)	678 (61.9)	158 (70.9)	759 (46)	602 (59.3)	93 (55)
Sex, No. (%)						
Male	540 (35.7)	538 (49.1)	128 (57.4)	484 (29.4)	588 (57.9)	134 (79.3)
Female	974 (64.3)	558 (50.9)	95 (42.6)	1165 (70.6)	427 (42.1)	35 (20.7)
Education, y	13.7 (2.2)	14.3 (2.2)	14.4 (2.0)	13.8 (2.1)	14.2 (2.2)	14.6 (2.1)
Currently smoking, No. (%)	389 (25.7)	290 (26.5)	44 (20)	438 (26.6)	252 (24.8)	33 (19.5)
Currently drinking alcohol, No. (%)	1260 (83.2)	969 (88.4)	201 (90)	1381 (83.7)	896 (88.3)	153 (90.5)
Alcohol intake, mL/d	128.5 (276.0)	168.9 (325.8)	215.5 (362.0)	119.4 (263.7)	186.7 (346.8)	243.9 (359.6)
Diet quality score	4.6 (0.9)	5.1 (1.0)	5.3 (1.1)	4.7 (0.9)	5.0 (1.0)	5.2 (1.1)
Total physical activity, EU	295.3 (221.8)	503.0 (276.4)	800.3 (367.4)	271.4 (189.5)	563.5 (266.8)	930.8 (333.4)
MPA, EU	95.4 (77.2)	175.7 (98.4)	294.0 (124.7)	112.1 (88.5)	174.3 (111.3)	242.0 (134.4)
VPA, EU	199.8 (184.5)	327.3 (228.5)	506.3 (294.5)	159.3 (139.3)	389.3 (207.5)	688.7 (261.9)
BMI	24.8 (5.3)	24.4 (4.5)	23.9 (3.9)	25.0 (5.5)	24.0 (4.0)	23.9 (3.3)
Waist circumference, cm	77.5 (11.8)	78.0 (10.6)	77.2 (10.2)	77.7 (12.1)	77.5 (10.0)	78.7 (9.2)
**Year 25**						
Age, y	50.0 (3.8)	50.2 (3.5)	50.3 (3.6)	50.1 (3.7)	50.1 (3.6)	50.0 (3.2)
Education, y	14.7 (2.7)	15.4 (2.6)	15.3 (2.4)	14.8 (2.7)	15.3 (2.6)	15.5 (2.7)
Currently smoking, No. (%)	296 (19.6)	263 (24.0)	29 (13)	339 (20.6)	236 (23.3)	28 (16.6)
Currently drinking alcohol, No. (%)	1074 (70.9)	886 (80.8)	188 (84.3)	1190 (72.2)	820 (80.8)	138 (81.7)
Alcohol intake, mL/d	97.7 (153.8)	130.0 (174.4)	131.6 (163.6)	90.7 (149.9)	138.7 (174.4)	167.0 (185.4)
Diet quality score	5.2 (1.0)	5.6 (0.9)	5.9 (1.0)	5.2 (0.9)	5.6 (1.0)	5.7 (1.0)
Total physical activity, EU	206.4 (187.7)	436.6 (260.4)	692.4 (308.1)	202.5 (165.6)	459.3 (244.1)	854.7 (325.8)
MPA, EU	75.3 (66.2)	181.0 (94.6)	302.2 (111.4)	102.1 (88.2)	167.6 (111.2)	243.3 (133.3)
VPA, EU	131.1 (160.6)	255.6 (223.3)	390.3 (258.6)	100.4 (119.4)	291.7 (192.2)	611.4 (275.2)
BMI	31.2 (7.6)	29.8 (6.7)	28.5 (5.6)	31.6 (7.7)	29.0 (6.1)	27.8 (5.1)
Waist circumference, cm	95.6 (16.5)	94.3 (15.6)	92.0 (14.1)	96.4 (16.6)	92.9 (15.1)	91.2 (13.5)
Liver attenuation, HU	56.0 (11.8)	54.9 (11.9)	54.9 (11.3)	55.3 (12.5)	55.8 (11.1)	55.3 (9.8)
<51, No. (%)	352 (23.2)	275 (25.1)	54 (24.2)	412 (25.0)	234 (23.1)	35 (20.7)
≤40, No. (%)	141 (9.3)	118 (10.8)	22 (9.9)	172 (10.4)	96 (9.5)	13 (7.7)

Abbreviations: BMI, body mass index (calculated as weight in kilograms divided by height in meters squared); EU, exercise units; HU, Hounsfield unit; MPA, moderate-intensity physical activity; VPA, vigorous-intensity physical activity.

^a^ All variables have less than 1% missing, except for diet quality score Y20 (21% missing).

### PA Trajectories

Three distinct MPA and VPA trajectories from young to middle adulthood were identified (Figure). In Figure A, 1514 participants (53.4%) maintained a very low stable and 1096 participants (38.7%) maintained a low increasing MPA trajectory over time, whereas 223 participants (7.9%) had a moderate increasing trajectory. The VPA trajectories showed that 1649 participants (58.2%) had a low stable and 1015 participants (35.8%) had a moderate decreasing trajectory, whereas 169 participants (6.0%) had a high decreasing trajectory (Figure B). Findings related to the association between total PA trajectories and NAFLD are presented in the eFigure, eTable 2, and eTable 3 in Supplement 1.

**Figure.**
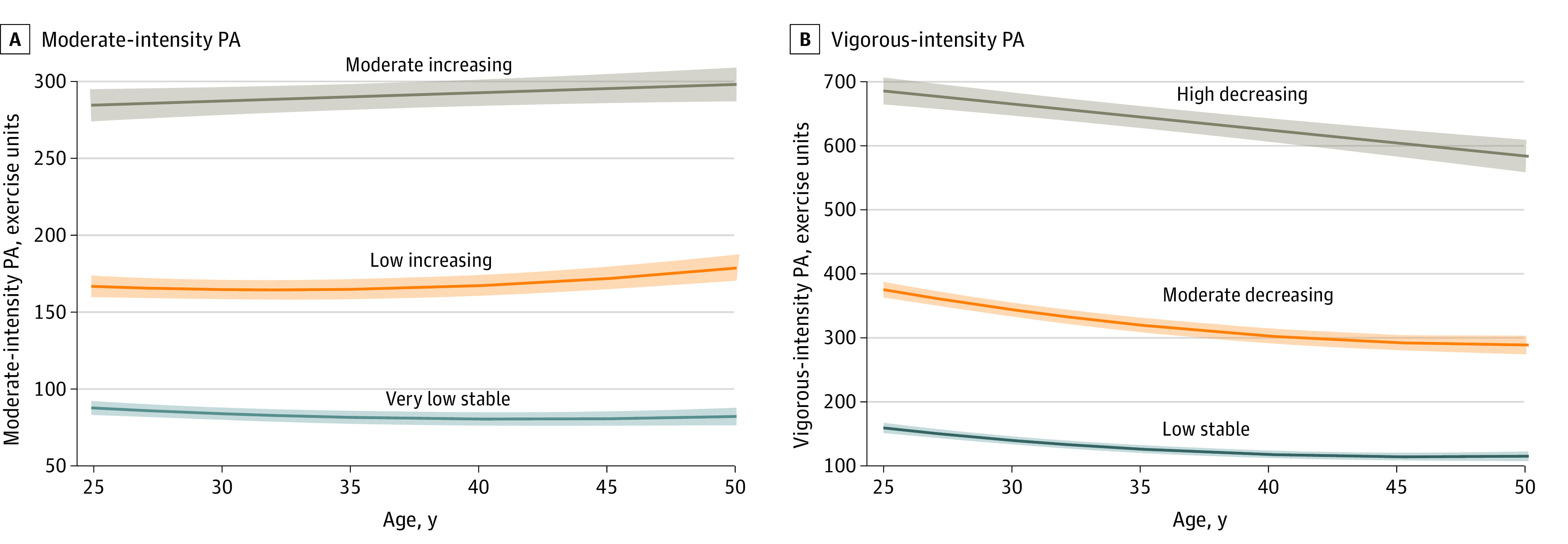
Moderate-Intensity and Vigorous-Intensity Physical Activity (PA) Trajectories From Young to Middle Adulthood in the Coronary Artery Risk Development in Young Adults Study (N = 2833) Shaded areas around trajectory lines indicate 95% CIs.

### PA Trajectories and NAFLD at LA Less Than 51

The associations between MPA and VPA trajectories and Y25 NAFLD at LA less than 51 are presented in Table 2 and Table 3, respectively. After adjustment for demographics (model 1) and further adjustment for education, and selected lifestyle risk factors (model 2), participants in the moderate decreasing (model 1: RR, 0.74; 95% CI, 0.64-0.85; model 2: RR, 0.74; 95% CI, 0.54-0.85) and the high decreasing (model 1: RR, 0.58; 95% CI, 0.43-0.79; model 2: RR, 0.59; 95% CI, 0.43-0.80) VPA trajectories had a lower risk of NAFLD compared with participants in the low stable VPA trajectory. Adjustments for baseline (Y0) BMI and waist circumference modestly attenuated these associations, but the results remained statistically significant (model 4 and model 5). The adjusted RRs across the MPA trajectories were close to the null, although the 95% CIs were consistent with either lower or higher risk of NAFLD. There was no evidence of effect modification by race and sex (results not presented).

**Table 2.** Unadjusted and Adjusted Associations Between Moderate-Intensity Physical Activity Trajectories From Young to Middle Adulthood and Nonalcoholic Fatty Liver Disease at Liver Attenuation Values Less Than 51 at Year 25^a^

	Moderate-intensity physical activity, RR (95% CI)		
	Very low stable	Low increasing	Moderate increasing
Unadjusted	1 [Reference]	1.08 (0.94-1.24)	1.04 (0.81-1.33)
Model 1	1 [Reference]	0.96 (0.84-1.10)	0.87 (0.68-1.11)
Model 2	1 [Reference]	0.97 (0.85-1.12)	0.88 (0.68. 1.12)
Model 3^b^	1 [Reference]	1.01 (0.86-1.18)	0.95 (0.72-1.24)
Model 4	1 [Reference]	0.97 (0.85-1.11)	0.91 (0.71-1.15)
Model 5	1 [Reference]	0.99 (0.87-1.14)	0.94 (0.74-1.19)

Abbreviation: RR, risk ratio.

^a^ Model 1 adjusted by sex, age, race, and study center. Model 2 adjusted by model 1 covariates and education, smoking status, and alcohol consumption. Model 3 adjusted by model 2 covariates and dietary intake. Model 4 adjusted by model 2 covariates and baseline (year 0) body mass index. Model 5 adjusted by model 2 covariates and baseline (year 0) waist circumference.

^b^ Total N = 2200 after the exclusion of participants with missing dietary intake.

**Table 3.** Unadjusted and Adjusted Associations Between Vigorous-Intensity Physical Activity Trajectories From Young to Middle Adulthood and Nonalcoholic Fatty Liver Disease at Liver Attenuation Values Less Than 51 at Year 25^a^

	Vigorous-intensity physical activity, RR (95% CI)		
	Low stable	Moderate decreasing	High decreasing
Unadjusted	1 [Reference]	0.92 (0.80-1.06)	0.83 (0.61-1.13)
Model 1	1 [Reference]	0.74 (0.64-0.85)	0.58 (0.43-0.79)
Model 2	1 [Reference]	0.74 (0.54-0.85)	0.59 (0.43-0.80)
Model 3^b^	1 [Reference]	0.80 (0.68-0.95)	0.66 (0.47-0.92)
Model 4	1 [Reference]	0.81 (0.70-0.93)	0.65 (0.48-0.88)
Model 5	1 [Reference]	0.84 (0.73-0.97)	0.68 (0.51-0.90)

Abbreviation: RR, risk ratio.

^a^ Model 1 adjusted by sex, age, race, and study center. Model 2 adjusted by model 1 covariates and education, smoking status, and alcohol consumption. Model 3 adjusted by model 2 covariates and dietary intake. Model 4 adjusted by model 2 covariates and baseline (year 0) body mass index. Model 5 adjusted by model 2 covariates and baseline (year 0) waist circumference.

^b^ Total N = 2200 after the exclusion of participants with missing dietary intake.

### PA Trajectories and NAFLD at LA Less Than or Equal to 40

After adjustment for demographics with model 1 (eTable 4 in Supplement 1), the risk of Y25 NAFLD at LA less than or equal to 40 was lower for participants in the moderate decreasing (RR, 0.73; 95% CI, 0.57-0.93) and high decreasing (RR, 0.53; 95% CI, 0.31-0.91) VPA trajectories relative to participants in the low stable VPA trajectory. These associations were attenuated and no longer significant in models 3 to 5 (eTable 4 in Supplement 1). We did not find evidence of effect modification by race and sex (results not presented).

## Discussion

In this prospective cohort study of Black and White adults, we identified 3 distinct trajectories of MPA and VPA over 25 years of follow-up. Our results found that participants following a high decreasing VPA trajectory had a 41% lower risk of NAFLD in middle adulthood compared with participants following a low stable pattern of VPA over time after adjusting for demographics and lifestyle risk factors. Adjustment for baseline BMI and waist circumference attenuated the observed associations. We did not find evidence of a lower risk of NAFLD for participants following moderate increasing or a low increasing MPA trajectories relative to those following a very low stable MPA trajectory in fully adjusted models. Although the RRs for the MPA trajectories did not suggest lower risk for NAFLD since they were close to the null, the lower limit of the 95% CIs indicated that a reduction of up to 30% in risk at higher MPA levels should not be ruled out. Overall, our findings suggest that sustaining higher levels of VPA from young adulthood to middle age could help reduce NAFLD risk, but more research on life course PA patterns and NAFLD is warranted.

To our knowledge, this is the first study that provides the long-term outcomes of PA trajectory patterns for midlife NAFLD risk. Modeling PA patterns over time helps identify groups of individuals with similar PA habits and provides a deeper understanding of the association of long-term PA with the risk of NAFLD. Our approach aligns with recommendations that stress the importance of a population-based approach to NAFLD prevention, as it recognizes that NAFLD etiology is a dynamic and ongoing process as people age and accumulate health issues.^25^

While this study did not find a direct association between MPA over the life course and NAFLD prevalence in midlife, it is important to note the many health benefits of MPA. The 2018 Physical Activity Guidelines for Americans suggest that adults engage in at least 150 minutes of MPA each week and that greater health benefits may be observed with more than 300 minutes of weekly PA.^12^ Given that our study found a potentially beneficial association between life course patterns of VPA and NAFLD in midlife among individuals who met or exceeded these PA guidelines, this suggests that more intense PA throughout the life course may be particularly beneficial in reducing the risk of NAFLD. Our findings are consistent with cross-sectional evidence linking higher PA intensities to a lower prevalence of NAFLD in adults.^26,27,28^ Moreover, our findings are aligned with several studies that have shown a dose-response relationship, where more intense exercise is further associated with improved insulin sensitivity,^29^ reduced oxidative stress and inflammation,^30^ and improved overall liver function by decreasing the activity of key enzymes involved in hepatic lipid synthesis,^31,32^ which could lead to a reduction in triglyceride accumulation and liver fat and an improvement in liver health.^33,34,35^

It is essential to recognize that VPA is not possible for some and challenging to maintain for most as people age. Thus, further research is needed to confirm our findings (ie, optimal PA intensity over the life course) and to better understand the association between PA patterns and NAFLD risk, including the optimal duration (ie, time spent in different types of PA) of PA for reducing NAFLD risk.

### Clinical Implications

The American Association for the Study of Liver Diseases recommends that to prevent or improve NAFLD, patients should engage in 150 minutes of MPA at least 5 times per week or increase their activity level by more than 60 minutes per week.^36^ While the optimal duration and intensity of PA should be tailored to each patient’s preferences and physical abilities, the guidance document emphasizes encouraging patients to exercise as much as possible.^36^ Our findings offer supporting evidence that higher PA intensities sustained over the life course may reduce the risk of NAFLD. That is, meeting (ie, ≥75 minutes/week equivalent to 300 EU) or exceeding by twice as much (ie, ≥150 minutes/week equivalent to 600 EU) the current VPA guidelines were associated with a lower risk of NAFLD in middle age.

To best assist patients with NAFLD, clinicians must consider the complex interplay of individual factors and structural and social determinants of health that may affect patients’ ability to make lifestyle changes,^37^ such as lower socioeconomic status and access to safe places to be physically active (eg, green spaces, fitness facilities),^38,39^ and engage in advocacy work aimed at achieving equitable access to care and treatments for all patients. Given known socioeconomic, racial, and ethnic disparities in NAFLD prevalence,^2,40^ clinicians and researchers need to approach the prevention and treatment of NAFLD with a health equity lens,^41^ recognizing the root causes (structural racism) and the social determinants of health that contribute to these disparities.^42^ Treatment interventions must be feasible and accessible to all patients, particularly for those who are disproportionately affected by these disparities.

### Limitations

Our findings should be interpreted in light of the study’s limitations. First, PA was assessed using a self-reported questionnaire, which is prone to measurement error (eg, recall bias) and social desirability bias. Second, because the CARDIA cohort recruited only Black and White participants, our results might not be generalizable to other racial and ethnic groups and populations with different sociodemographic characteristics, particularly those with the highest prevalence of NAFLD, such as Hispanic populations and those with low socioeconomic status.^2,40^ Future trials and population-based cohort studies should include proper representation of the populations that are most affected by NAFLD to help develop future guidelines that support equitable access to prevention and treatment of NAFLD for all patients.^41^ Third, our findings are unable to differentiate incident from prevalent NAFLD, as only a single assessment of NAFLD was evaluated. While it is unknown whether participants had NAFLD at baseline, CARDIA began in 1985 to 1986 before the rapid rise in obesity and diabetes prevalence when participants were at a mean age of 25 years, thus with a likely low overall prevalence of NAFLD at baseline. Additionally, we acknowledge the recent nomenclature shift from NAFLD to metabolic dysfunction–associated steatotic liver disease (MASLD).^43^ Although we used NAFLD criteria in our study, we believe that most of our participants would meet criteria for MASLD.^44,45^

## Conclusions

This cohort study’s results suggest that individuals who met or exceeded the current 2018 Physical Activity Guidelines for Americans (ie, ≥75 minutes/week for moderate intensity and ≥150 minutes/week for vigorous intensity) from young to middle adulthood had a lower risk of NAFLD in middle age compared with those who engaged in less than the current recommendations. Targeted delivery of equitable prevention programs aimed at modifying lifestyle risk factors over the life course, particularly facilitating VPA, may be important to reduce NAFLD risk in midlife.

## Notes

Supplement 1. **eTable 1.** Average Posterior Probability by Physical Activity Trajectories **eFigure.** Total Physical Activity Trajectories **eTable 2.** Selected Baseline (Year 0) and Year 25 Participant Characteristics by Total Physical Activity Trajectory Groups (N= 2833) **eTable 3.** Unadjusted and Adjusted Associations Between Total Physical Activity Trajectories from Young to Middle Adulthood and NAFLD at LA <51 at Year 25 **eTable 4.** Unadjusted and Adjusted Associations Between Moderate-Intensity and Vigorous-Intensity Physical Activity Trajectories from Young to Middle Adulthood and NAFLD at LA ≤40 at Year 25

Supplement 2.
Data Sharing Statement
