# Supplementary material for: Young Adult Physical Activity Trajectories and Midlife Nonalcoholic Fatty Liver Disease
Source: JAMA Netw Open. 2023 Oct 20;6(10):e2338952. doi: 10.1001/jamanetworkopen.2023.38952 (PMC10589812; doi:10.1001/jamanetworkopen.2023.38952)
Supplement: Supplement 1. — eTable 1. Average Posterior Probability by Physical Activity Trajectories eFigure. Total Physical Activity Trajectories eTable 2. Selected Baseline (Year 0) and Year 25 Participant Characteristics by Total Physical Activity Trajectory Groups (N= 2833) eTable 3. Unadjusted and Adjusted Associations Between Total Physical Activity Trajectories from Young to Middle Adulthood and NAFLD at LA <51 at Year 25 eTable 4. Unadjusted and Adjusted Associations Between Moderate-Intensity and Vigorous-Intensity Physical Activity Trajectories from Young to Middle Adulthood and NAFLD at LA ≤40 at Year 25 [file jamanetwopen-e2338952-s001.pdf]

Supplemental Online Content

de Brito JN, McDonough DJ, Mathew M, et al. Young adult physical activity trajectories and midlife nonalcoholic fatty liver disease. *JAMA Netw Open.* 2023;6(10):e2338952. doi:10.1001/jamanetworkopen.2023.38952

eTable 1. Average Posterior Probability by Physical Activity Trajectories

eFigure. Total Physical Activity Trajectories

eTable 2. Selected Baseline (Year 0) and Year 25 Participant Characteristics by Total Physical Activity Trajectory Groups (N= 2833)

eTable 3. Unadjusted and Adjusted Associations Between Total Physical Activity Trajectories from Young to Middle Adulthood and NAFLD at LA <51 at Year 25

eTable 4. Unadjusted and Adjusted Associations Between Moderate-Intensity and Vigorous-Intensity Physical Activity Trajectories from Young to Middle Adulthood and NAFLD at LA ≤40 at Year 25

This supplemental material has been provided by the authors to give readers additional information about their work

© 2023 de Brito JN et al. *JAMA Network Open.*

eTable 1. Average Posterior Probability by Physical Activity Trajectories.

Physical Activity Trajectories	Average posterior probability	Proportion with posterior probabilities ≥0.70
Total Physical Activity		
Very low stable	1.0	1.0
Moderate stable	0.9	1.0
High decreasing	0.9	1.0
Moderate-Intensity Physical Activity		
Very low stable	0.9	1.0
Low increasing	0.9	1.0
Moderate increasing	0.9	1.0
Vigorous-Intensity Physical Activity		
Low stable	0.9	1.0
Moderate decreasing	0.9	1.0
High decreasing	0.9	1.0

eTable 2. Selected Baseline (Year 0) and Year 25 Participant Characteristics by Total Physical Activity Trajectory Groups (N= 2833)

	**Total Physical Activity Trajectories**		
	Low stable (n = 1496) mean ± SD	Moderate stable (n = 1032) mean ± SD	High decreasing (n = 305) mean ± SD
Baseline (Year 0)			
Age, y, mean (SD)	24.9 ± 3.7	25.0 ± 3.5	25.0 ± 3.3
Race, n (%)			
Black	924 (56)	371 (38)	84 (42)
White	725 (44)	615 (62)	114 (58)
Sex, n (%)			
Male	498 (30)	554 (56)	154 (78)
Female	1151 (70)	432 (44)	44 (22)
Education, y	13.7 ± 2.1	14.3 ± 2.2	14.4 ± 2.2
Current smoker, n (%)	437 (29)	246 (24)	40 (13)
Alcohol drinker, n (%)	1379 (92)	873 (85)	178 (58)
Alcohol intake, mL/day, mean (SD)	116.3 ± 256.5	194.2 ± 358.0	224.3 ± 342.6
Diet quality score	4.7 ± 0.9	5.1 ± 1.0	5.2 ± 1.1
Total physical activity, EU	263.9 ± 181.0	566.7 ± 252.2	923.9 ± 342.9
MPA, EU	103.5 ± 81.0	181.9 ± 106.2	265.7 ± 138.5
VPA, EU	160.4 ± 140.4	384.8 ± 205.2	658.2 ± 274.5
BMI, kg/m^2	25.0 ± 5.5	24.0 ± 4.0	24.0 ± 3.3
Waist circumference, cm	77.6 ± 12.1	77.5 ± 9.9	79.0 ± 9.3
Year 25			
Age, y	50.0 ± 3.7	50.2 ± 3.6	50.1 ± 3.3
Education, y	14.8 ± 2.7	15.4 ± 2.6	15.2 ± 2.6
Current smoker, n (%)	235 (16)	237 (23)	31 (10)
Alcohol drinker, n (%)	1183 (79)	807 (78)	158 (52)
Alcohol intake, mL/day	91.8 ± 153.8	136.3 ± 168.6	165.0 ± 188.6
Diet quality score	5.2 ± 0.9	5.6 ± 0.9	5.7 ± 1.0
Total physical activity, EU	196.1 ± 159.8	463.2 ± 237.0	829.9 ± 316.3
MPA, EU	93.3 ± 80.3	175.3 ± 107.4	266.7 ± 128.8
VPA, EU	102.8 ± 122.6	287.9 ± 195.4	563.3 ± 279.9
BMI, kg/m^2	31.6 ± 7.7	29.1 ± 6.2	28.2 ± 5.2
Waist circumference, cm	96.2 ± 16.6	93.0 ± 15.1	92.4 ± 13.5
Liver attenuation, HU	55.5 ± 12.3	55.6 ± 11.3	54.5 ± 10.9
< 51, n (%)	411 (27)	222 (21)	48 (16)
≤ 40, n (%)	168 (11)	93 (9)	20 (7)

Abbreviations: EU, exercise units; MPA, moderate-intensity physical activity; VPA, vigorous-intensity physical activity; BMI, Body Mass Index, HU, Hounsfield Units

[a] All variables have less than 1% missing, except for diet quality score Y20 (21% missing).

© 2023 de Brito JN et al. *JAMA Network Open.*

eFigure. Total Physical Activity Trajectories

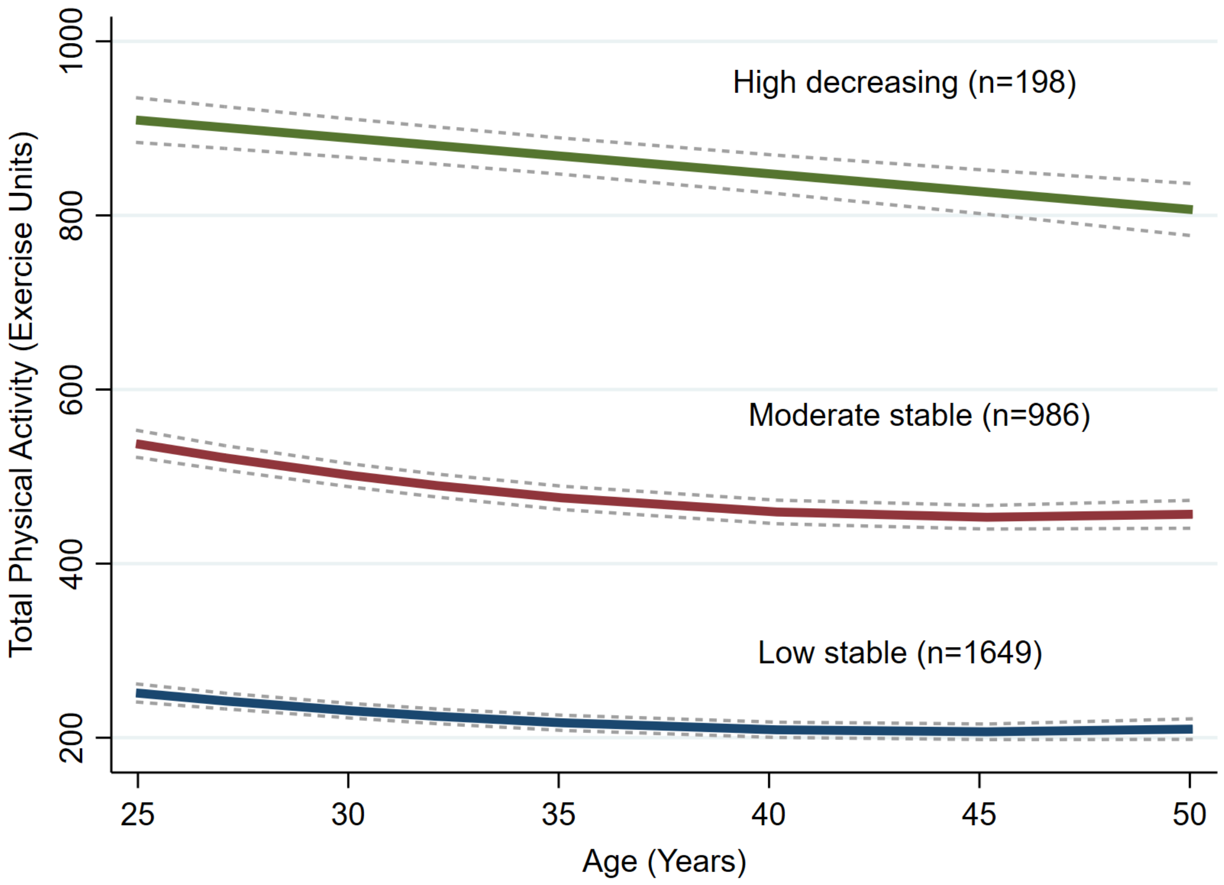

eTable 3. Unadjusted and Adjusted Associations Between Total Physical Activity Trajectories from Young to Middle Adulthood and NAFLD at LA <51 HU at Year 25

**Total Physical Activity**	Low stable		Moderate stable		High decreasing	
	RR	95% CI	RR	95% CI	RR	95% CI
Unadjusted	Reference		0.90	(0.78, 1.04)	0.97	(0.75, 1.26)
Model 1			0.73	(0.63, 0.84)	0.69	(0.53, 0.89)
Model 2			0.73	(0.63, 0.85)	0.70	(0.54, 0.91)
Model 3[a]			0.75	(0.64, 0.88)	0.75	(0.56, 1.01)
Model 4			0.79	(0.68, 0.91)	0.76	(0.59, 0.98)
Model 5			0.81	(0.71, 0.94)	0.78	(0.61, 1.01)

Abbreviations: NAFLD, non-alcoholic fatty liver disease; LA, liver attenuation; HU, Hounsfield Unit

Model 1 adjusted by sex, age, race, and study center. Model 2 adjusted by Model 1 covariates and education, smoking status, and alcohol consumption. Model 3 adjusted by Model 2 covariates and dietary intake. Model 4 adjusted by Model 2 covariates and Y0 BMI. Model 5 adjusted by Model 2 covariates and Y0 waist circumference.

[a] Total N = 2200 after the exclusion of participants with missing dietary intake.

© 2023 de Brito JN et al. *JAMA Network Open.*

eTable 4. Unadjusted and Adjusted Associations Between Moderate-Intensity and Vigorous-Intensity Physical Activity Trajectories from Young to Middle Adulthood and NAFLD at LA ≤40 at Year 25

Total Physical Activity

	Low stable		Moderate stable		High decreasing	
	RR	95% CI	RR	95% CI	RR	95% CI
Unadjusted	Reference		0.93	(0.73, 1.18)	0.99	(0.64, 1.53)
Model 1			0.74	(0.58, 0.95)	0.71	(0.45, 1.11)
Model 2			0.75	(0.58, 0.95)	0.71	(0.45, 1.13)
Model 3[a]			0.79	(0.60, 1.03)	0.81	(0.49, 1.34)
Model 4			0.80	(0.62, 1.24)	0.78	(0.50, 1.24)
Model 5			0.84	(0.65, 1.09)	0.82	(0.53, 1.29)

Moderate-Intensity Physical Activity

	Very low stable		Low increasing		Moderate increasing	
	RR	95% CI	RR	95% CI	RR	95% CI
Unadjusted	Reference		1.16	(0.92, 1.46)	1.06	(0.69, 1.62)
Model 1			1.00	(0.79, 1.27)	0.85	(0.55, 1.30)
Model 2			1.02	(0.80, 1.31)	0.84	(0.54, 1.31)
Model 3[a]			1.10	(0.84, 1.44)	1.02	(0.64, 1.62)
Model 4			1.02	(0.80, 1.30)	0.89	(0.56, 1.36)
Model 5			1.05	(0.83, 1.34)	0.91	(0.59, 1.42)

Vigorous-Intensity Physical Activity

	Low stable		Moderate decreasing		High decreasing	
	RR	95% CI	RR	95% CI	RR	95% CI
Unadjusted	Reference		0.91	(0.71, 1.15)	0.74	(0.43, 1.27)
Model 1			0.73	(0.57, 0.93)	0.53	(0.31, 0.92)
Model 2			0.73	(0.58, 0.91)	0.51	(0.29, 0.91)
Model 3[a]			0.83	(0.63, 1.09)	0.55	(0.29, 1.04)
Model 4			0.81	(0.63, 1.04)	0.58	(0.33, 1.02)
Model 5			0.85	(0.67, 1.10)	0.61	(0.35, 1.06)

Abbreviations: NAFLD, non-alcoholic fatty liver disease; LA, liver attenuation; HU, Hounsfield Unit

Model 1 adjusted by sex, age, race, and study center. Model 2 adjusted by Model 1 covariates and education, smoking status, and alcohol consumption. Model 3 adjusted by Model 2 covariates and dietary intake. Model 4 adjusted by Model 2 covariate and Y0 BMI. Model 5 adjusted by Model 2 covariates and Y0 waist circumference.

[a] Total N = 2200 after the exclusion of participants with missing dietary intake.

© 2023 de Brito JN et al. *JAMA Network Open.*
