# Supplementary material for: Young Adult Physical Activity Trajectories and Midlife Nonalcoholic Fatty Liver Disease
Source: JAMA Netw Open. 2023 Oct 20;6(10):e2338952. doi: 10.1001/jamanetworkopen.2023.38952 (PMC10589812; doi:10.1001/jamanetworkopen.2023.38952)
Supplement: Supplement 2. — Data Sharing Statement [file jamanetwopen-e2338952-s002.pdf]

Data Sharing Statement

de Brito. Young Adult Physical Activity Trajectories and Midlife Nonalcoholic Fatty Liver Disease. *JAMA Netw Open*. Published October 20, 2023.
doi:10.1001/jamanetworkopen.2023.38952

Data
Data available: No
